# Synergistic effect of a novel autophagy inhibitor and Quizartinib enhances cancer cell death

**DOI:** 10.1038/s41419-017-0170-9

**Published:** 2018-01-26

**Authors:** Amanda Tomie Ouchida, Yingbo Li, Jiefei Geng, Ayaz Najafov, Dimitry Ofengeim, Xiaoxiao Sun, Qiang Yu, Junying Yuan

**Affiliations:** 1 Department of Cell Biology, Harvard Medical School, 240 Longwood Ave., Boston, MA 02115 USA; 2 Ludwig Cancer Center, Harvard Medical School, Boston, MA 02115 USA; 3 Shanghai Institute of Materia Medica, Chinese Academy of Sciences, 555 Zuchongzhi Road, Shanghai, 201203 China

## Abstract

Drug combinations have been increasingly applied in chemotherapy as a strategy to enhance the efficacy of anti-cancer treatment. The appropriate drug combinations may achieve synergistic effects beyond monotherapies alone. AC220 (Quizartinib), an FLT3 receptor tyrosine kinase inhibitor, developed for the treatment of AML, has been tested in phase II human clinical trials. However, AC220 as a monotherapy is not efficacious enough. In this study, we performed a small-molecule screening of 12 640 compounds in order to find a compound that increase the AC220 efficacy in chemotherapy. We identified that TAK-165, a HER2 inhibitor, even when used at low nanomolar doses in combination with AC220, was able to induce cell death in different cancer cells, but not in non-cancer cell lines. We showed that TAK-165 and AC220 act synergistically to downregulate key signaling pathways and potently induce cancer cell death. Furthermore, we demonstrated that TAK-165 inhibited autophagy in a HER2-independent manner. Finally, we showed that the combination of TAK-165 and AC220 induced cell death in cancer cells through the activation of chaperone-mediated autophagy. Overall, these findings support the strategy for using AC220 and an autophagy inhibitor such as TAK-165 in a combinatorial treatment to enhance the efficacy of cancer therapies.

## Introduction

FLT3, a member of receptor tyrosine kinase III family, is highly expressed in normal bone marrow cells, early progenitor cells and hematopoietic stem cells. FLT3 stimulation promotes cell proliferation by activating phosphoinositol-3-kinase (PI3K), Ras GTPase, protein kinase B (Akt) and mitogen-activated protein kinase (MAPK) pathways^1^. Cancer-related FLT3 mutations in leukemia, especially acute myeloid leukemia (AML), can induce ligand-independent activation of the receptor and promote proliferation of hematological tumor cells^2–4^. Thus, FLT3 has been recognized as a promising target in AML chemotherapy. AC220 (also called Quizartinib), a potent and selective inhibitor of FLT3, was developed for AML treatment and had been tested in phase II human clinical trials^5^. AC220 was shown to be a highly specific for FLT3 in a kinome profiling experiment^6^. In addition, AC220 has demonstrated acceptable pharmacokinetic properties and pharmacokinetic profile, as well as efficacy and tolerability in xenographic tumor models and in humans^6,7^. Although the early clinical studies have shown promising outcomes for AC220 as a monotherapy, cancer recurrence in AML patients treated with AC220 has suggested difficulty in using AC220 as monotherapy. AC220 in combination with other chemotherapeutic agents has been shown to improve disease recurrence rates in AML^7–9^. The use of AC220 in other types of cancers has not been well-explored.

Autophagy is an evolutionarily conserved mechanism that functions to promote the degradation and recycling of cellular components through lysosomes^10–12^. Autophagy is activated in eukaryotic cells as an adaptive and survival mechanism in response to stress and starvation in order to maintain cellular homeostasis. Autophagy activation has been shown to be an important regulator of cancer development and progression and thus, inhibition of autophagy has been considered as a possible anti-cancer therapy, such as in combination therapies with the use of chemotherapeutic agents that can inhibit autophagy^13–15^. Consistently, inhibition of autophagy has been shown to decrease tumor growth, as activation of autophagy can protect against genotoxic stress^13^. Here we screened the ICCB Known Bioactive library of 12,640 compounds for the enhancement of the cytotoxicity of AC220 and identified TAK-165, a potent and irreversible HER2 (encoded by *ERBB2*) inhibitor, as a compound that can effectively induce cell death in different cancer cell lines when used in combined therapy with AC220. We demonstrated that TAK-165 could act as an autophagy inhibitor by a HER2-independent mechanism and induce cell death through chaperone-mediated autophagy when used in combination with AC220. The identification of TAK-165 as an autophagy inhibitor and its high efficacy in killing cancer cells when combined with AC220 suggests the possibility of inhibiting autophagy in a combination therapy for combating resistance to cancer treatment.

## Results

### TAK-165 in a combinatorial treatment with AC220 induces cell death in different cancer cell lines

**Fig. 1:**
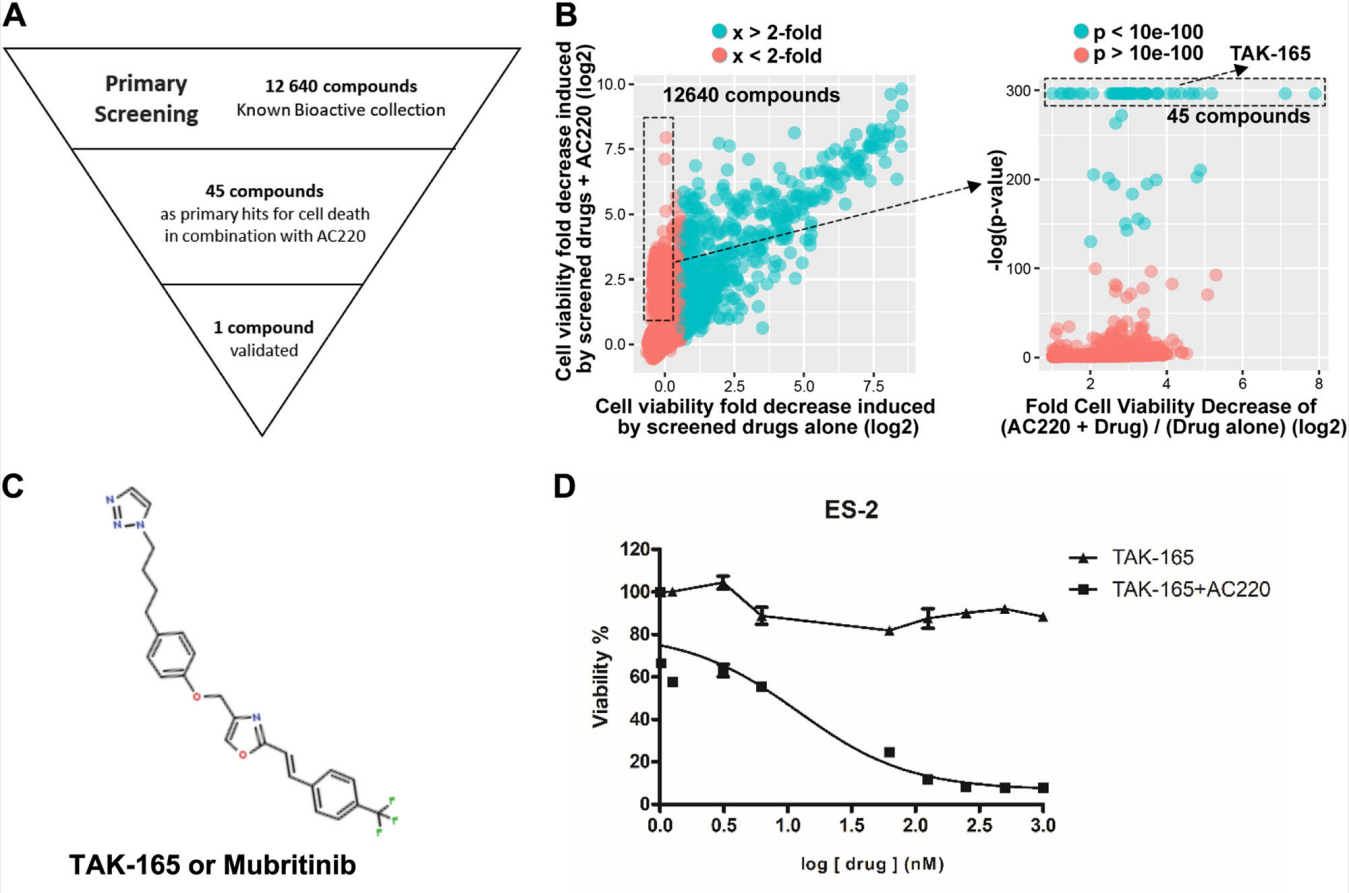
Identification of TAK-165 as a compound that increases the cytotoxicity of AC220 in ES-2 cells **a** Outline of high-throughput screen of cytotoxicity of 12,640 compounds with or without AC220 in ES-2 cells. **b** Left panel: Fold cell survival data for the screened compounds with (y-axis) or without (x-axis) AC220, with respect to untreated control. Right panel: Student’s *t* test with respect to untreated control showing the most statistically significant hits. TAK-165 was discovered among the top 45 hits that did not induce cell death alone, but induced cell death in combination with AC220. **c** TAK-165 (Mubritinib) chemical structure. **d** Dose-response curve of TAK-165 alone and in combination with AC220 in ES-2 cells. ES-2 cells were treated with TAK-165 at indicated concentrations and AC220 at 2 μM for 24 h. Viability was determined using CellTiter-Glo® Luminescent assay (*n* = 3). Bars: Mean ± SD. *p < 0.05; **p < 0.01; ***p < 0.001

**Fig. 2:**
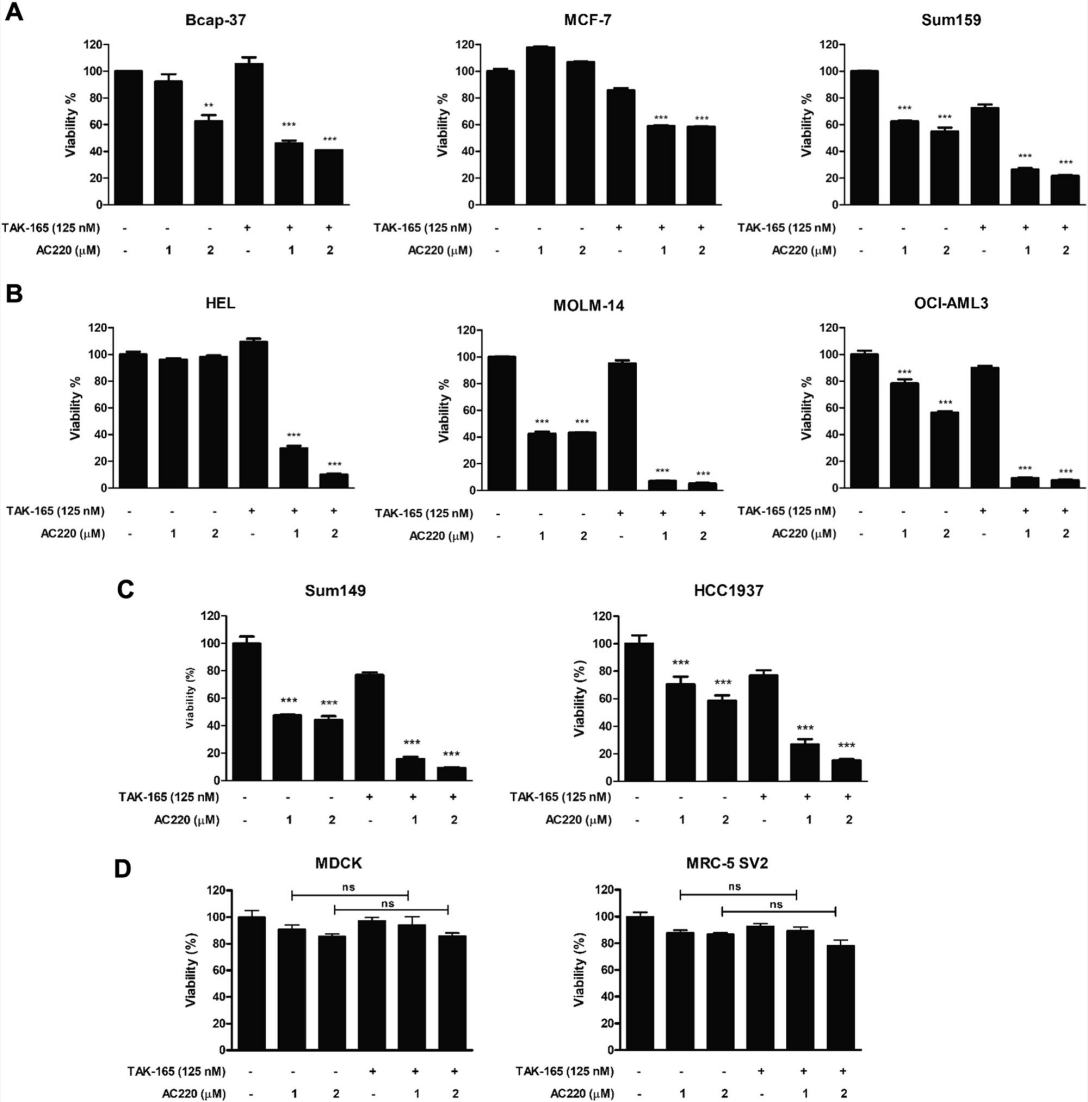
TAK-165 in combination with AC220 induces cell death in breast, AML, and breast triple negative cancer cell lines **a** Cell viability (%) of breast cancer (Bcap-37, MCF-7 and Sum159), **b** AML (HEL, MOLM-14 and OCI-AML3), **c** triple negative breast cancer (Sum149 and HCC1937), and **d** non-cancer cell lines (MDCK and MRC-5 SV2) treated with TAK-165 (125 nM) and AC220 at 2 different concentrations (1 and 2 μM) for 24 h. Viability was determined using Sulforhodamine B assay (Breast cancer cells) and CellTiter-Glo® Luminescent assay (AML, breast triple negative and non-cancer cell lines). In all the experiments, treatment groups were compared with control group, unless otherwise indicated (*n* = 3). Bars: Mean ± SD. *p < 0.05; **p < 0.01; ***p < 0.001

In order to find a compound that could enhance AC220 efficacy in chemotherapy, we performed a small-molecule screening of 12,640 compounds from the ICCB Known Bioactive library. This high-throughput screening was performed on ES-2 cells, which express FLT3 (ref^16^.), using a luminescence-based viability assay. From the primary screening, 45 primary hits were obtained (Fig. 1a, b), and after validations in secondary assays, TAK-165 was identified (Fig. 1c) as a compound that was able to induce cell death when combined with AC220. TAK-165 was originally identified as a selective, irreversible and potent HER2 inhibitor^17^. TAK-165 demonstrated no cytotoxicity in ES-2 cells when used as a single treatment, however, it was able to reduce cell viability in a dose- and time-dependent manner when treated in combination with AC220 (Fig. 1d and Supplementary Fig. 1A). We also tested whether TAK-165/AC220 combination could affect the viability of other cancer cell lines, including those derived from AML and breast cancer. We found that both breast cancer cell lines (BCAP-37, MCF-7 and Sum159) as well as AML cell lines (HEL, MOLM-14 and OCI-AML3) showed significant reduction of viability when treated with the combination TAK-165/AC220 (Fig. 2a, b, Supplementary Fig. 1B and 1C). The triple negative breast cancer cell lines (Sum149 and HCC1937), which are negative for estrogen receptors, progesterone receptors, and HER2^18^, also showed a significant loss of viability after treatment with TAK-165 and AC220 (Fig. 2c). On the other hand, the TAK-165/AC220 combination did not show a significant effect on viability of non-cancer cell lines, as MDCK and MRC-5 SV2 (Fig. 2d). This suggests that the TAK-165/AC220 combination may be promising for the treatment of different types of cancer.

### TAK-165 has a synergistic effect with AC220 in inhibiting proliferation signaling

**Fig. 3:**
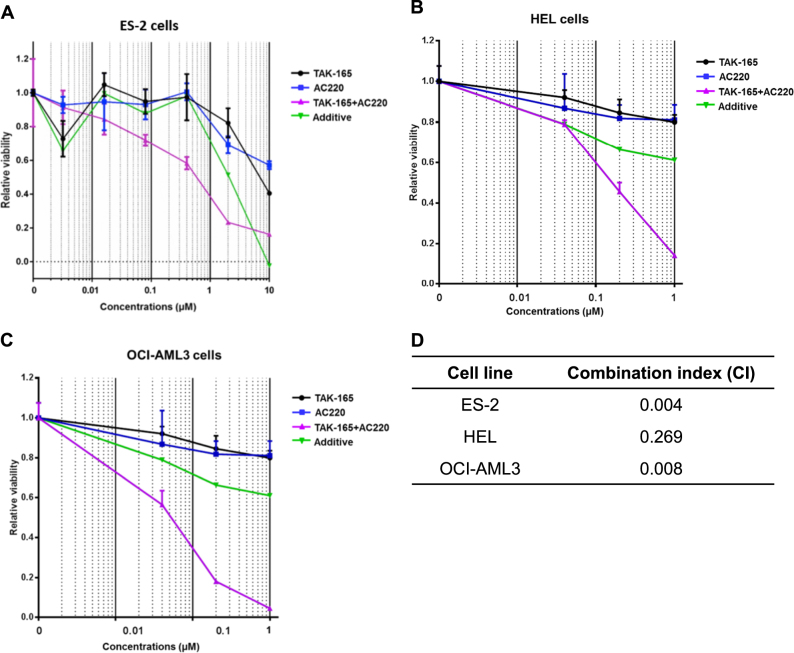
TAK-165 synergizes with AC220 to kill cancer cells Combination between TAK-165 and AC220 showed a synergistic effects in cancer cell lines. **a** ES-2 (**b**) HEL and (**c**) OCI-AML-3 were exposed to TAK-165, AC220 or a combination of TAK-165 plus AC220 at three or four different concentrations (0.01, 0.1, 1 and 10 μM) for 24 h and analyzed by MTT. **d** The combination index was determined by CompuSyn software using the results of MTT

The combination index may be used to determine if a drug combination may have additive or synergistic effects^19–21^. We determined the combination index between the TAK-165 and AC220, in order to assess whether the viability loss in tumor cells by the combination treatment may have synergistic action between these two compounds. We found that the combination index of combined treatment with TAK-165 and AC220 on ES-2 cells (Fig. 3a) and on two AML cell lines, HEL (Fig. 3b) and OCI-AML3 (Fig. 3c) all showed a strong combination index of below 0.3 (Fig. 3d), suggesting that TAK-165 has a very strong synergistic effect with AC220 in promoting the cytotoxicity.

**Fig. 4:**
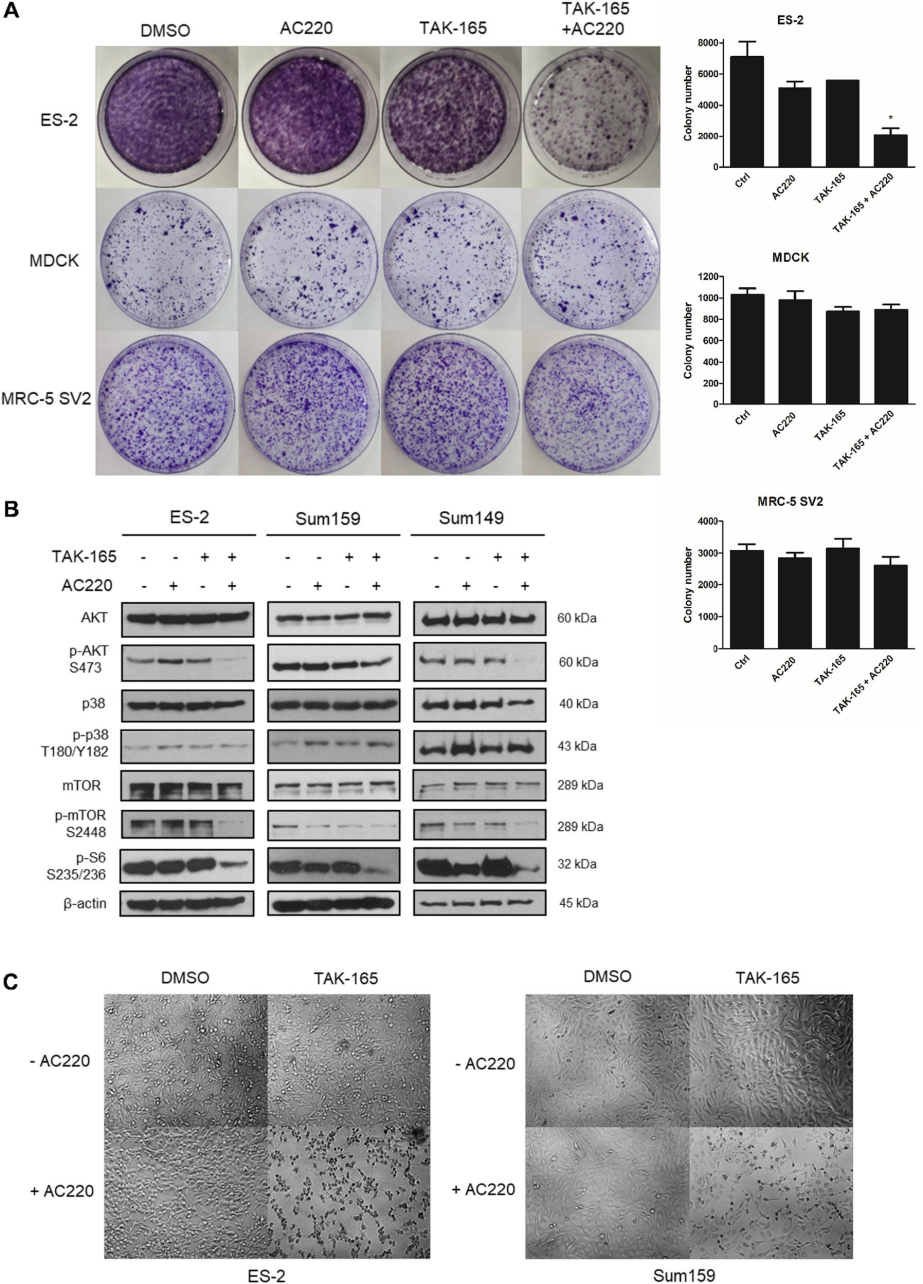
TAK-165/AC220 combination affects cellular proliferation of cancer cells **a** TAK-165 in combination with AC220 suppressed the growth of ovarian cancer cells but not of non-cancer cells. Colony formation assay for ES-2, MDCK and MRC-5 SV2 cells treated with TAK-165 (125 nM) and AC220 (2 μM) for 24 h. Right panel shows the quantitative analysis of the colony forming assay. The colony number was determined by ImageJ. **b** Immunoblotting of phospho- and total Akt, p38, mTOR and pS6 levels of ES-2, Sum159 and Sum149 treated with TAK-165 (125 nM) and AC220 (2 μM) for 2 h. Anti-β-actin was used as a loading control. **c** TAK-165/AC220 combination treatments reduces viability of ES-2 and Sum159 cells in a non-proliferative state (at 100% confluence). ES-2 and Sum159 cells were treated with TAK-165 (125 nM) and AC220 (2 μM) for 24 h and visualized under a phase-contrast microscope (magnification: 200×)

Since AC220 is an FLT3 inhibitor, while TAK-165 is a HER2 inhibitor, and both of these kinases mediated cellular pathways are involved in mediating cell proliferation signals^6,22,23^, we next tested whether the TAK-165/AC220 combination treatment could affect cell proliferation. We found that TAK-165/AC220 combination was able to reduce colony formation in ES-2 cells, but not in MDCK and MRC-5 SV2 cells (Fig. 4a). Since both FLT3 and HER2 are involved in regulating the activation of the Akt/mTOR complex I (mTORC1) signaling pathway, we next compared the effect of single and combination treatment of TAK-165 and AC220 in ES-2, Sum159 and Sum149 cells on the activation of this pathway. We found that TAK-165/AC220 combination was much more effective than treatment with either compound alone in reducing the phosphorylation of Akt Ser473 site and mTOR Ser2448 site, the biomarkers of their activation, as well as that of S6, which is downstream of mTORC1 signaling, in ES-2 (ovarian cancer), Sum159 (breast cancer) and Sum149 (triple negative breast cancer) cell lines (Fig. 4b). On the other hand, the activation of p38 MAPK, which is important for mediating cellular stress^24^, was unaltered after combination treatment in all cell lines (Fig. 4b). These data suggest that both FLT3 and HER2 may be involved in mediating the activation of Akt and mTORC1 in these cells and inhibition of either pathway alone is not sufficient to block their growth signaling; while combined TAK-165/AC220 treatment led to inactivation of Akt/mTORC1 pathway, but not to alternative pathways such as p38 MAPK.

Next, we evaluated whether the effect of TAK-165/AC220 combination was dependent upon cell proliferation by treating confluent and therefore non-proliferative cells, since the cell death induction in this type of condition is considered to be one of the challenges for chemotherapies^25^. We plated ES-2 cells and Sum159 cells in confluent condition and after TAK-165/AC220 combination treatment for 24 h, we analyzed cell morphology using phase-contrast microscopy. We found that both cell lines showed rounded up and necrosis-like morphology after combined treatment, suggesting that they were dying (Fig. 4c). This observation was confirmed by a cell death assay, which showed a significant loss of cell viability in both ES-2 and Sum159 cells in a dose-dependent manner after TAK-165/AC220 combination treatment (Supplementary Figure 2A).

### TAK-165 inhibits autophagy in a HER2-independent manner

**Fig. 5:**
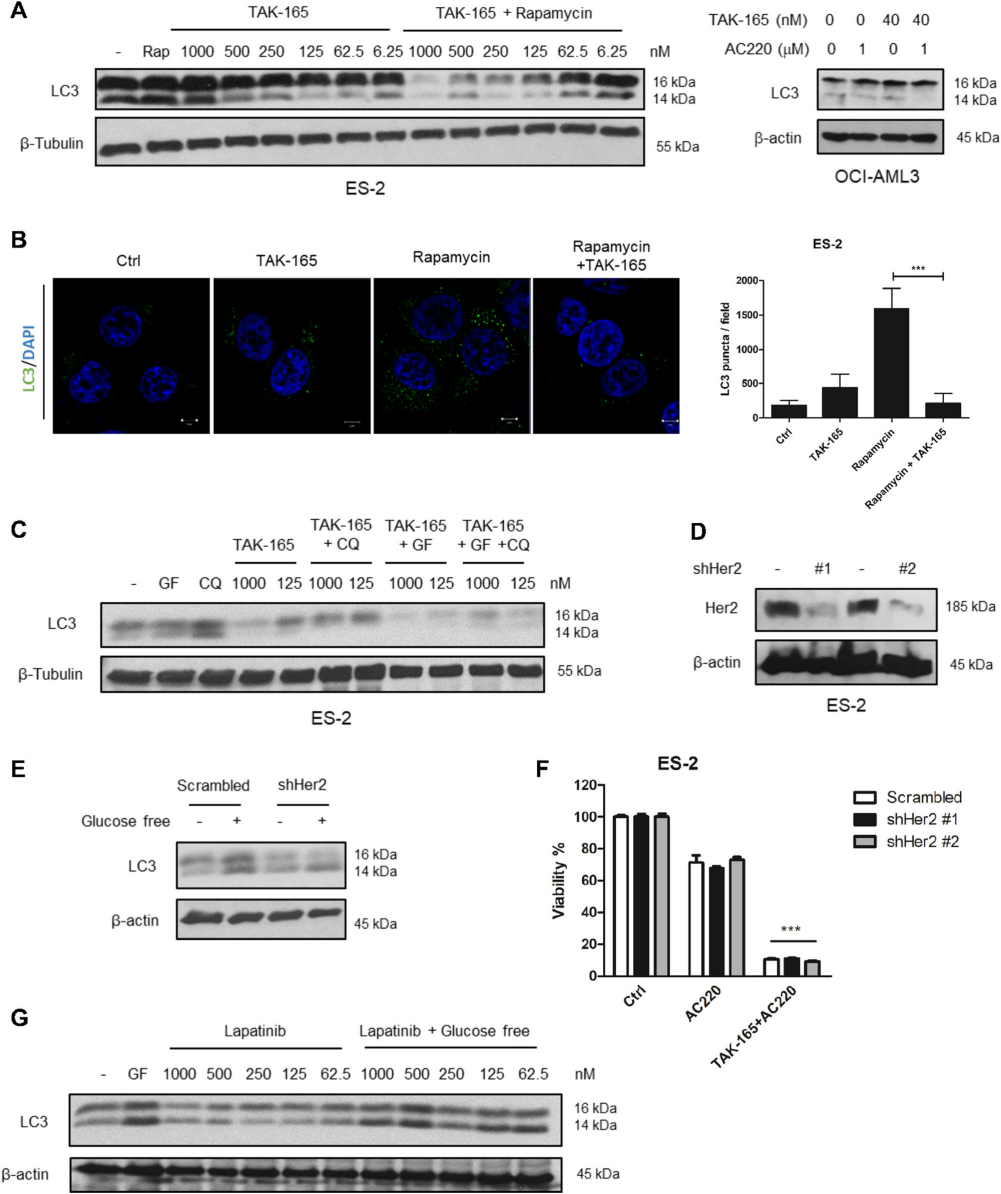
TAK-165 inhibits autophagy in a HER2-independent manner **a** Immunoblotting of LC3 protein levels in ES-2 and OCI-AML3 cells treated with decreasing concentrations of TAK-165 and/or 200 nM Rapamycin (Rap) for 16 h (right), or treated in combination with 1 μM AC220 for 16 h (left). **b** Immunofluorescence staining of LC3. ES-2 cells treated with TAK-165 (1 μM) and/or Rapamycin (200 nM) for 16 h were immunostained for LC3 (green) and counterstained with DAPI (blue) to localize the nucleus (right). Images were captured using a 63x objective of a laser scanning confocal microscope. Scale bar: 5 μm. LC3 puncta were quantified by ImageJ software, using at least five different images from two separate experiments (left). **c** Analysis of autophagic flux of ES-2 cells. Immunoblotting of LC3 protein levels in ES-2 cells, treated with 2 different concentrations of TAK-165 (1000 and 125 nM) and/or 50 μM Chloroquine (CQ - lysosomal inhibitor) for 16 h, in presence or absence of glucose-free (GF) conditions. **d** HER2 does not inhibit autophagy. Immunoblotting of HER2 and (**e**) LC3 protein levels in ES-2 stables cell lines for HER2 knockdown, in presence or absence of glucose-free conditions for 16 h. **f** Cell viability (%) of ES-2 stable cell lines for HER2 knockdown treated with AC220 (2 μM) or a combination of TAK-165 (125 nM) plus AC220 for 24 h. Viability was determined using CellTiter-Glo® Luminescent assay (*n* = 3). **g** Immunoblotting of LC3 protein levels in ES-2 treated with decreasing concentrations of Lapatinib and/or glucose-free (GF) conditions for 16 h. Anti-β-tubulin and anti-β-actin were used as a loading control. In all the experiments, treatment groups were compared with control group, unless otherwise indicated. Bars: Mean ± SD. ***: *p* < 0.001

We have previously shown that the combination of AC220 with Spautin-1, an autophagy inhibitor, is able to induce cell death in cancer cells^16^. Thus, to investigate the mechanism of cell death caused by the TAK-165/AC220 combination, we next evaluated whether TAK-165 has any effect on autophagy. Interestingly, we found that TAK-165 was able to reduce LC3 lipidation in a dose-dependent manner when stimulated by Rapamycin, an mTORC1 inhibitor, in ES-2 cells (Fig. 5a, left) or by AC220, which mimics glucose-free conditions, in HEL cells (Fig. 5a, right). Moreover, TAK-165 reduced LC3 puncta formation upon autophagy induction by Rapamycin treatment in ES-2 cells (Fig. 5b). These experiments suggest that TAK-165 can potently inhibit LC3 lipidation, a critical step in autophagy. We also assessed whether treatment with TAK-165 would be reducing autophagic flux. As shown in Fig. 5c, after treatment with the lysosomal inhibitor Chloroquine, TAK-165 (at two different concentrations), and upon autophagy stimulation by glucose-free conditions, we observed a reduction of LC3 lipidation, demonstrating that TAK-165 inhibits autophagic flux under both basal level and glucose-free conditions.

Since TAK-165 is a HER2 inhibitor^17^, we next evaluated whether the autophagy inhibition induced by TAK-165 is due to HER2 inhibition. We performed HER2 knockdown in ES-2 cells using two different shRNAs (Fig. 5d). However, HER2 knockdown (shHER2) was not able to inhibit autophagy in ES-2 cells under glucose-free condition (Fig. 5e). Similar to the scramble clone, both shHer2 clones were sensitive to TAK-165/AC220 combination while the loss of viability was not observed when exposed to AC220 alone (Fig. 5f). In addition, we also evaluated whether the use of Lapatinib, another known HER2 inhibitor, would be able to inhibit autophagy. As shown in Fig. 5g, no effect on LC3 lipidation was observed following Lapatinib treatment in glucose-free condition. Furthermore, Lapatinib and AC220 combination was not able to induce cell death in ES-2 cells (Supplementary Fig. 3A). Overall, these data suggest that TAK-165 can inhibit autophagy in a HER2-independent manner.

### Activation of chaperone-mediated autophagy by TAK-165/AC220 combination induces cell death

**Fig. 6:**
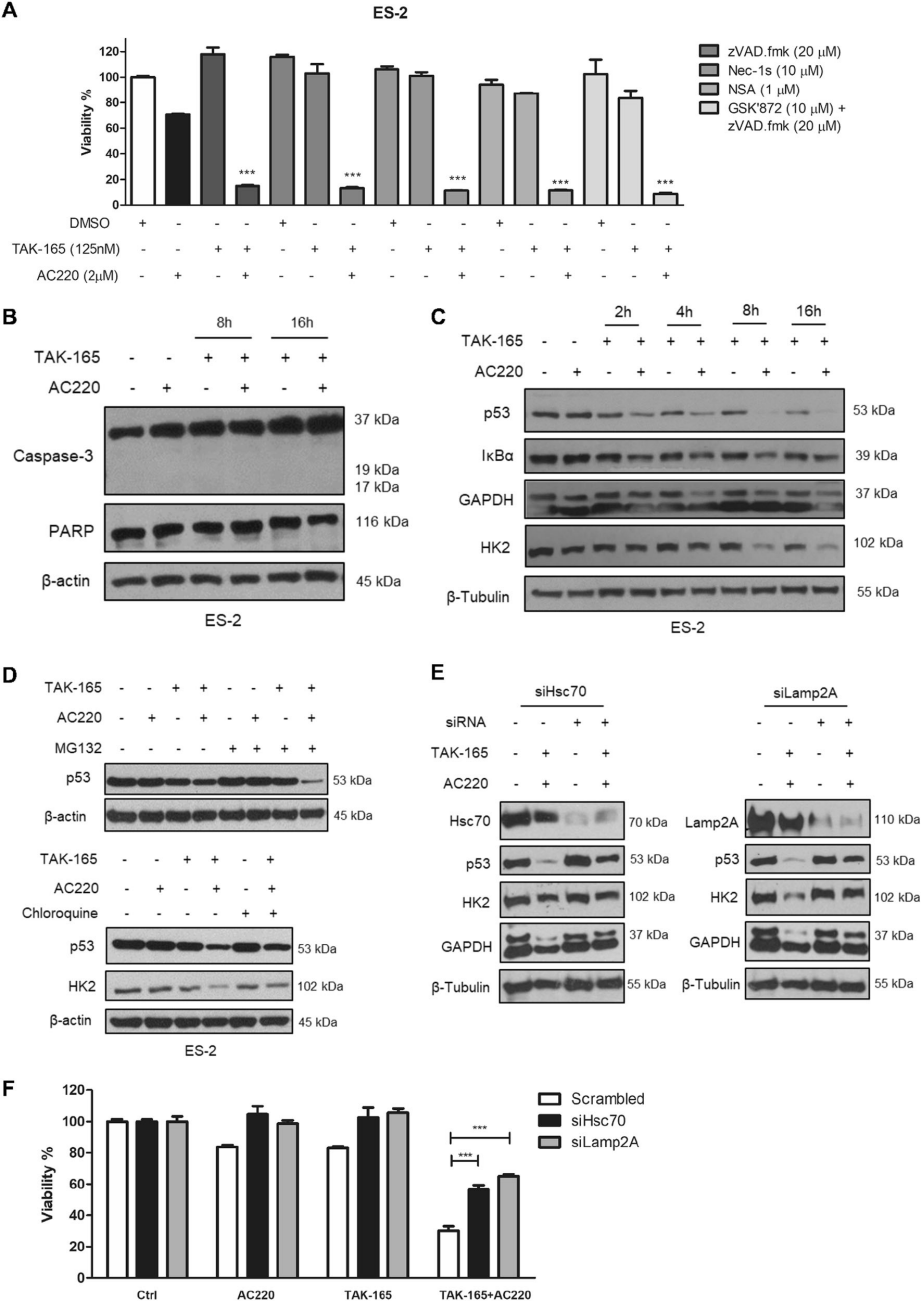
TAK-165/AC220 induces cell death through Chaperone-mediated autophagy activation **a** Cell viability (%) of ES-2 cells treated with treated with TAK-165, AC220 or a combination of TAK-165 plus AC220 in the presence or absence of zVAD.fmk, Nec-1s, NSA and GSK832 for 24 h (*n* = 3). Viability was determined using CellTiter-Glo® Luminescent assay. **b** Immunoblotting of caspase-3 and PARP-1 in ES-2 cells treated with TAK-165 (125 nM) and/or AC220 (2 μM) for 8 and 16 h. **c** TAK-165/AC220 combination activates chaperone-mediated autophagy. Immunoblotting of p53, IκB-α, GAPDH and HK2 levels in ES-2 cells treated with TAK-165 and/or AC220 up to 16 h. **d** Immunoblotting of p53 levels in ES-2 cells treated with TAK-165 and AC220 for 6 h in the absence or presence of MG132 (10 μM, proteasome inhibitor) and Chloroquine (CQ, 50 μM). **e** Immunoblotting of Lamp2A, Hsc70, p53, HK2 and GAPDH levels in Scramble, Hsc70, or Lamp2A siRNA-transfected ES-2 cells treated with TAK-165 (125 nM) and AC220 (2 μM) for 8 h. **f** Cell viability (%) of scramble, Lamp2A, or Hsc70 siRNA-transfected ES-2 cells treated with TAK-165 and/or AC220 for 8 h. Viability was determined using CellTiter-Glo® Luminescent assay (*n* = 3). Anti–β-tubulin and anti-β-actin were used as a loading control. In all the experiments, treatment groups were compared with control group, unless otherwise indicated. Bars: Mean ± SD. ***p < 0.001

To investigate the mechanism of cell death induced by TAK-165/AC220 combination, we employed zVAD.fmk, a pan-caspase inhibitor that strongly blocks apoptotic cell death; as well as three necroptosis inhibitors: Nec-1s (RIPK1 inhibitor), necrosulfonamide (NSA, MLKL inhibitor) and GSK’872 (RIPK3 inhibitor). As shown in Fig. 6a, none of these inhibitors prevented the loss of viability induced by TAK-165/AC220 combination and no caspase-3 or PARP cleavage was detected after 8 or 16 h of treatment (Fig. 6b). These data indicate that TAK-165/AC220 combination induces neither apoptosis nor necroptosis.

Since we have shown that autophagy inhibition by Spautin-1 in combination with AC220 lead to chaperone-mediated autophagy (CMA) activation and consequent cell death of cancer cells^16^, we next explored whether the combination TAK-165/AC220 also induces CMA. As observed in Fig. 6c, the combinatorial treatment between TAK-165 and AC220 induced the degradation of different CMA substrates, including mutant p53, IκB-α, GAPDH and HK2 in a time-dependent manner in ES-2 cells. CMA activation was also observed in other cell lines such as breast cancer cells (MDA-MB-231, Sum159 and MDA-MB-435), and an AML line (HEL, Supplementary Fig. 4A). To determine whether the degradation of these proteins is mediated through lysosomal or proteasomal pathways, we used the protease inhibitor MG132 or the lysosomal inhibitor chloroquine (CQ) concomitantly with TAK-165/AC220 combination and examined the levels of mutant p53 as a biomarker for CMA activation. As shown in Fig. 6d, while the treatment of MG132 further reduced the levels of mutant p53, CQ treatment recovered mutant p53 levels when used concomitantly with TAK-165/AC220 combination. These results suggest that TAK-165/AC220 combination induces the degradation of mutant p53 through lysosomal pathway.

Hsc70 and Lamp2A are two key mediators of CMA^26–28^. To test the involvement of CMA, we used siRNAs to knockdown Hsc70 (siHsc70) and Lamp2A (siLamp2A). We observed a blockage in reduction of mutant p53, HK2, and GAPDH levels after TAK-165 and AC220 combinatorial treatment (Fig. 6e), as well as a partial recovery of cell viability of ES-2 cells upon combined treatment of TAK-165 and AC220 (Fig. 6f). These results suggest that the cytotoxicity of combined TAK-165 and AC220 treatment is at least in part mediated through CMA. To investigate whether CMA activation would be connected to HER2 inhibition, we used Lapatinib along with AC220 and evaluated mutant p53 levels. We did not observe mutant p53 degradation upon Lapatinib and AC220 combined treatment (Supplementary Fig. 4B), which suggests that the CMA induction is also HER2 independent. Thus, these data suggest that the TAK-165/AC220 combinatorial treatment induces cell death in different types of cancer through CMA activation independent of HER2.

## Discussion

Resistance of cancers to chemotherapeutics is a key reason that leads to the failure of cancer treatment^29,30^. One important strategy to overcome cancer resistance to chemotherapy is through the use of combinatorial treatments, since it is possible to use two different drugs that inhibit the key signaling pathway in different ways, thereby blocking the activation of potential resistance mechanisms; or by the use of two or more drugs that target different signaling pathways, leading to impairment of cancer cell survival^31–33^. AC220 is a second-generation class III FLT3 inhibitor that has been tested in phase II human clinical trials, however, it has been shown that AC220 may be not sufficiently efficacious as a monotherapy^6,7^. In this study, we performed a small-molecule compound screening to identify potential drugs that increase the chemotherapeutic efficacy of AC220. Interestingly, we identified TAK-165 as a new autophagy inhibitor that can induce cell death in different types of cancer, but not in non-cancer cells, when combined with AC220.

TAK-165 (Mubritinib) is a potent, selective and irreversible HER2 receptor tyrosine kinase inhibitor that was tested in phase I human clinical trials^17,23^. HER2 is a transmembrane receptor tyrosine kinase that is commonly overexpressed in breast cancer cells and promotes cell proliferation and survival^22^. Thereby, HER2 inhibition blocks these signaling pathways to promote cytotoxicity in cancer cells^22,34,35^. Our results demonstrate that TAK-165 treatment at nanomolar concentrations concomitantly with AC220 reduces the viability of ovarian cancer (ES-2), breast cancer (Sum159, Bcap-37 and MCF-7), AML (HEL, OCI-AML3 and MOLM-14), as well as triple negative breast cancer cell lines (Sum149 and HCC1937), suggesting that an off-target effect of TAK-165^36^ mediates its synergistic action with AC220, since triple negative breast cancer cells do not express HER2^37^(Fig. 2 and Supplementary Figure 1). These data suggest that TAK-165 can work to synergistically improve AC220 efficacy in several types of cancer cell lines independent of HER2.

The comparison between normal tissues and solid tumors shows that tumors tend to exhibit high cell density due to rapid growth and high proliferative activity of cancer cells, which might in part contribute to the resistance to chemotherapeutic treatment^38–40^. Our results demonstrate that the TAK-165/AC220 combination is able to induce cell death in both ES-2 and Sum159 cells, in a confluent and non-proliferative state (Fig. 4c). In addition, we have found that TAK-165/AC220 combination affects cell proliferation of cancer cells, but not that of non-cancer cells, such as MDCK and MRC-5 SV2 (Fig. 4a). Moreover, the TAK-165/AC220 combination also potently inhibits the activation of key intracellular signaling pathways (Fig. 4b), including Akt and mTORC1^41–44^. In summary, our data suggest the use of TAK-165 and AC220 is able to induce cell death in both proliferative and non-proliferative conditions.

Classic autophagy inhibitors may show characteristic reduction of LC3-II, indicating a blockage in the lipidation of LC3-I^45–47^. Such examples include class III PI3K inhibitors, such as 3-methyladenine, Wortmannin and LY294002^45,48^, which can inhibit autophagy in early stages. On other hand, CQ, Hydroxychloroquine, and Bafilomycin A inhibit autophagy at late stages by suppressing the lysosomal function^49–51^. Our results demonstrate that TAK-165 is able to reduce LC3-I lipidation upon autophagy activation by combined treatment with Rapamycin or in glucose-free conditions, which inhibit mTOR and mimic nutrient deprivation, respectively^52,53^. In addition, treatment with CQ and glucose-free conditions shows that TAK-165 reduces the autophagic flux (Fig. 5c). Furthermore, autophagy inhibition induced by TAK-165 is triggered independently of HER2 (Fig. 5d, e), reinforcing that TAK-165 has an off-target effect that can inhibit autophagy.

Our data also demonstrate that TAK-165 and AC220 act synergistically in the induction of cell death in different cell lines (Fig. 3). Although all cancer cells tested have shown a strong combination index (CI), HEL and OCI-AML3 cells, both AML cells, presented distinct CI values. This fact might be due to the differential sensitivity of these cells to TAK-165 and/or AC220 alone treatment. Other previous studies have demonstrated that AC220 acts synergistically with other compounds inducing cell death, as follows: (i) the histone deacetylase inhibitor Panabinostat;^54^ (ii) the DNA methyltransferase inhibitor 5-azacitidine;^9^ (iii) receptor tyrosine kinase inhibitors, such as Dasatinib^55^ and Sorafenib^56^, and (iv) JQ1, a BET protein antagonist, a transcriptional regulatory protein^57^. However, this is the first report of AC220 acting synergistically with an autophagy inhibitor, suggesting the possibility of using other autophagy inhibitors in combination with AC220 to enhance its efficacy in chemotherapy.

The TAK-165/AC220 combination potently activates CMA, as evidenced by the degradation of known CMA substrates, including mutant p53, GAPDH, IκB-α and Hexokinase-II (Fig. 6c and Supplementary Fig. 4)^16,28,58,59^. In addition, both cell viability and degradation of these substrates are recovered upon Hsc70 and Lamp2A knockdown, as well as by CQ lysosomal inhibition (Figs. 6d, e, f), suggesting that the activation of CMA is at least in part responsible for the cytotoxicity of combination treatment. CMA promotes the degradation of proteins individually selected by the chaperone-recognition motif in their amino acid sequence and delivered directly to the lysosome for degradation. Thus, CMA allows the removal of specific proteins and makes it an efficient system for degradation of damaged or abnormal proteins^26,60,61^. In addition, the malfunction of CMA may contribute to cancers;^62^ however, the activation of this pathway may induce an increase in proliferation^63^, or even cell death^16,59,64,65^, demonstrating that CMA may play a double role in cancer. Previously, our group have shown that Spautin-1/AC220 combination also induces CMA in cancer cells^16^. However, Spautin-1 may have cardiotoxicity in mice^59^. Thus, the use of TAK-165 in a combinatorial treatment may have potential value, since TAK-165 had been tested in a phase I human clinical trials and is able to inhibit autophagy at nanomolar concentrations. Therefore, in this study, we identified for the first time the compound TAK-165 as an autophagy inhibitor, which promotes cell death in different cancer cells through CMA activation when treated in combination with AC220, that promotes metabolic stress and a strong dependence of autophagy for cell survival^16^. Further studies should be performed in order to clarify about the mechanism of action as well as off-target effects of TAK-165, since our treatment strategy demonstrates its potential in combinatorial treatment.

## Material and methods

### Cell culture and treatment conditions

Cells lines were cultured as described previously^16^. The triple negative cell lines, Sum149 and HCC1937, were maintained in RPMI-1640 supplemented with 10% fetal bovine serum (FBS), penicillin (100 U/ml), and streptomycin (100 mg/ml) at 37 °C in a humidified atmosphere containing 5% CO_2_. The non-cancer cell line MRC-5 SV2 was maintained in DMEM supplemented with 10% FBS, penicillin (100 U/ml), streptomycin (100 mg/ml), 2 mM Glutamine and 1% MEM Non-essential Amino Acid Solution at same conditions described above. Treatments were performed either at non-confluent conditions (~60% confluence) or 100% confluent conditions. For glucose-free conditions, cell culture media with no glucose was supplemented with dialyzed FBS. TAK-165 (125 nM; Selleck Chemicals, Texas, USA) and AC220 (2 μM; Selleck Chemicals) were used unless otherwise stated. Lapatinib (125 nM; Sigma-Aldrich, Missouri, USA), zVAD.fmk (20 μM, Sigma-Aldrich), Nec-1s (R-7-Cl-O-Nec-1–10 μM), NSA (Necrosulfonamide, 1 μM, Sigma-Aldrich) or GSK’872 (10 μM, Millipore, Massachusetts, USA) co-treatment was used to inhibit apoptosis or necroptosis. DMSO (0.1%) was used as a control. For autophagy induction, Rapamycin (200 nM; Sigma-Aldrich) was used. Chloroquine (50 μM; Sigma-Aldrich) and MG132 (10 μM; Sigma-Aldrich) were added in the last 4 h of the treatment. For cells in non-proliferative state, phase-contrast imaging was performed for each cell line after combination treatment for 24 h. A series of digital images were acquired to cover the entire well plate using a Nikon Eclipse Ti-E microscope (Japan).

### Generation of ES-2 shHER2 cells and Hsc70 and Lamp2A knockdown

pLKO.1puro lentiviral plasmid (Sigma-Aldrich) was used to generate ES-2 cells that stably express the short-hairpin RNA against human HER2 (NC_000017) or non-targeting shRNA. The two target sequences for shRNA against human HER2 were: clone 1 (TRCN0000039878) 5′-GTGTCAGTATCCAGGCTTTGTA-3′, and clone 2 (TRCN0000039882) 5′-GGAATATGTGAACCAGCCAGAT-3′. The non-targeting sequence of shRNA (SHC002), which targets no known mammalian genes, is 5′-CAACAAGATGAAGAGCACCAA-3′. shRNA lentivirus and cell generation were prepared as previously described^66^. For Hsc70 and Lamp2A knockdown, ES-2 cells were transfected with siRNA against Hsc70 and Lamp2A (GenPharma, China), as well as scrambled siRNA (GenPharma, China), as negative control, using LipoFectamine RNAiMAX reagent (Thermo Fisher Scientific, Massachusetts, USA) according to the manufacturer’s instructions. After 48–72 h, knockdown efficiency was monitored by immunoblotting analysis.

### Chemical library screening

The screening for compounds that enhance AC220 efficiency as chemotherapeutic agent was performed in ES-2 cells seeded in 384-well plates under non-confluent condition 24 h prior to the compound transfer. A total of 12,640 compounds from the ICCB Known Bioactive Libraries were individually transferred to the wells, and AC220 (2 μM) was added next to all the wells. After incubating for 24 h, CellTiter-Glo® Luminescent Cell Viability assay (Promega, Wincosin, USA) was performed. Primary hits were based on *Z* scores calculated using the formula *Z* = (X − Ave_Neg)/ SD_Neg or more than 70% of difference in cell death between library alone and in combination with AC220. In all, 45 compounds were identified from the primary screening and cherry-picked for a secondary screen. The plates were screened in duplicates. Spautin-1 (C43) (10 μM) and AC220 (2 μM) in combination were used as a positive control for cell death.

### Sulforhodamin B assay and CellTiter-Glo® Luminescent viability assay

Sulforhodamine B (SRB) viability assay was performed as described previously^67^. Briefly, cells were seeded in 96-well plates 24 h prior to treatment. The cells were treated with different concentrations of TAK-165 and AC220, as described above. After 24 h of incubation, the plates were washed with 1x PBS twice. Then the cells were fixed with ice-cold 3.3% TCA at 4 °C for 1 h. After incubation, the plates were washed thoroughly with water and dried at room temperature. SRB solution (0.057%) was added to the plates and incubated at room temperature for 30 min. The plates were then washed with 0.01% acetic acid solution and dried at room temperature. Tris solution (10 mM pH 10.5) was added in each well and the plates were shaken on an orbital shaker for 5 min at room temperature. Absorbance was determined at 510 nm using a Biotek (Vermont, USA) microplate reader. CellTiter-Glo® Luminescent viability assay was also used to determine cell viability according to the manufacturer’s instructions.

### Immunoblotting and antibodies used

Cells were collected and lysed using RIPA buffer (150 mM NaCl, 1% IGEPAL® CA-630, 0.5% sodium deoxycholate, 0.1% SDS and 50 mM Tris-HCl pH 8) supplemented with protease and phosphatase inhibitors (Roche, Switzerland) and total protein concentration were measured by BCA assay. Whole-cell lysates (~20–30 μg of proteins) were separated by SDS-PAGE and transferred to a nitrocellulose membrane. Immunoblotting was performed using the following primary antibodies: anti-p38 (#8690), anti-phospho p38 (T180/Y182 - #4511), anti-p53 (#2524), anti-phospho pS6 (S235/236 - #4058), anti-Akt (#9272), anti-phospho-Akt (S473, #4060), anti-caspase-3 (clone 8G10; #9665), anti-HK2 (#2867), anti-HER2 (#2165), anti-mTOR (#2983), anti-phospho-mTOR (S2448—#5536), anti-PARP (#9542) from Cell Signaling Technology (Massachusetts, USA); anti-LC3 (L7543) from Sigma-Aldrich; anti-Actin (sc-8432), anti-GAPDH (sc-166545), anti-IκB-α (sc-371), anti-Lamp2 (sc-18822) and anti-tubulin (sc-73242) from Santa Cruz Biotechnology (Texas, USA); and anti-Hsc70 (10654–1-AP) from Proteintech (Illinois, USA).

### Immunofluorescence for LC3 puncta formation assay

Cells were seeded in glass coverslips in 24-well plate and treated as described above for 16 h. Samples were fixed with 4% paraformaldehyde (Sigma-Aldrich) for 30 min at room temperature. LC3 primary antibody (L7543, Sigma-Aldrich) was incubated overnight. Cells were washed 3 times with PBS-T, incubated with an Alexa Fluor 488-conjugated secondary antibody for 1 h, counterstained with DAPI (Thermo Fisher Scientific). Cells were visualized in a Confocal LSM 710 microscope Zeiss (Germany), and analyzed by Zen Lite software (Zeiss). The number of LC3 puncta/field was determined by an ImageJ software version 1.47t.

### Colony formation assay

Cells were seeded in 6-well plates and treated as described above for 24 h. These cells were counted, and reseeded in 100 mm plates (1000 cells per plate) and maintained in incubator at 37 °C in a humidified atmosphere containing 5% CO_2_ for 14 days. Culture media were changed each 4 days. The cells were then fixed with 4% paraformaldehyde for 15 min at room temperature. The fixed cellular monolayer was washed with 1× PBS, and 0.1% crystal violet was added and incubated for 20 min at room temperature. After incubation time, the plates were washed with 1× PBS and dried at room temperature. The plates were scanned in HP scanjet G2710 scanner and analyzed using ImageJ software version 1.47t.

### Combination index determination

Cells were seeded in a 96-well plate 24 h prior to treatment. In the following day, cells were treated with TAK-165 at concentrations from 10 to 0.05 μM in combination with AC220 at concentrations from 10 to 0.05 μM. After 24 h of incubation, MTT assay was performed as described previously^68^ to measure cell viability. The method described by Chou-Talalay^69^ was used to determine the combination index (CI) by using CompuSyn software. CI < 1, = 1, and >1 indicate synergism, additive effect, and antagonism, respectively.

### Statistical analysis

GraphPad Prism version 5.01 software was used for statistical analysis. The data were represented as mean ± SD. *P* values less than 0.05 were considered statistically significant (*p < 0.05; **p < 0.01; ***p < 0.001) and one-way ANOVA (Tukey’s Multiple Comparison Test) was used for all analysis.

## Electronic supplementary material

Supplementary Figures 1, 2, 3 and 4

Supplementary Figures Legends
