# Supplementary material for: Synergistic effect of a novel autophagy inhibitor and Quizartinib enhances cancer cell death
Source: Cell Death Dis. 2018 Jan 26;9(2):138. doi: 10.1038/s41419-017-0170-9 (PMC5833862; doi:10.1038/s41419-017-0170-9)

# Supplementary Figure 1

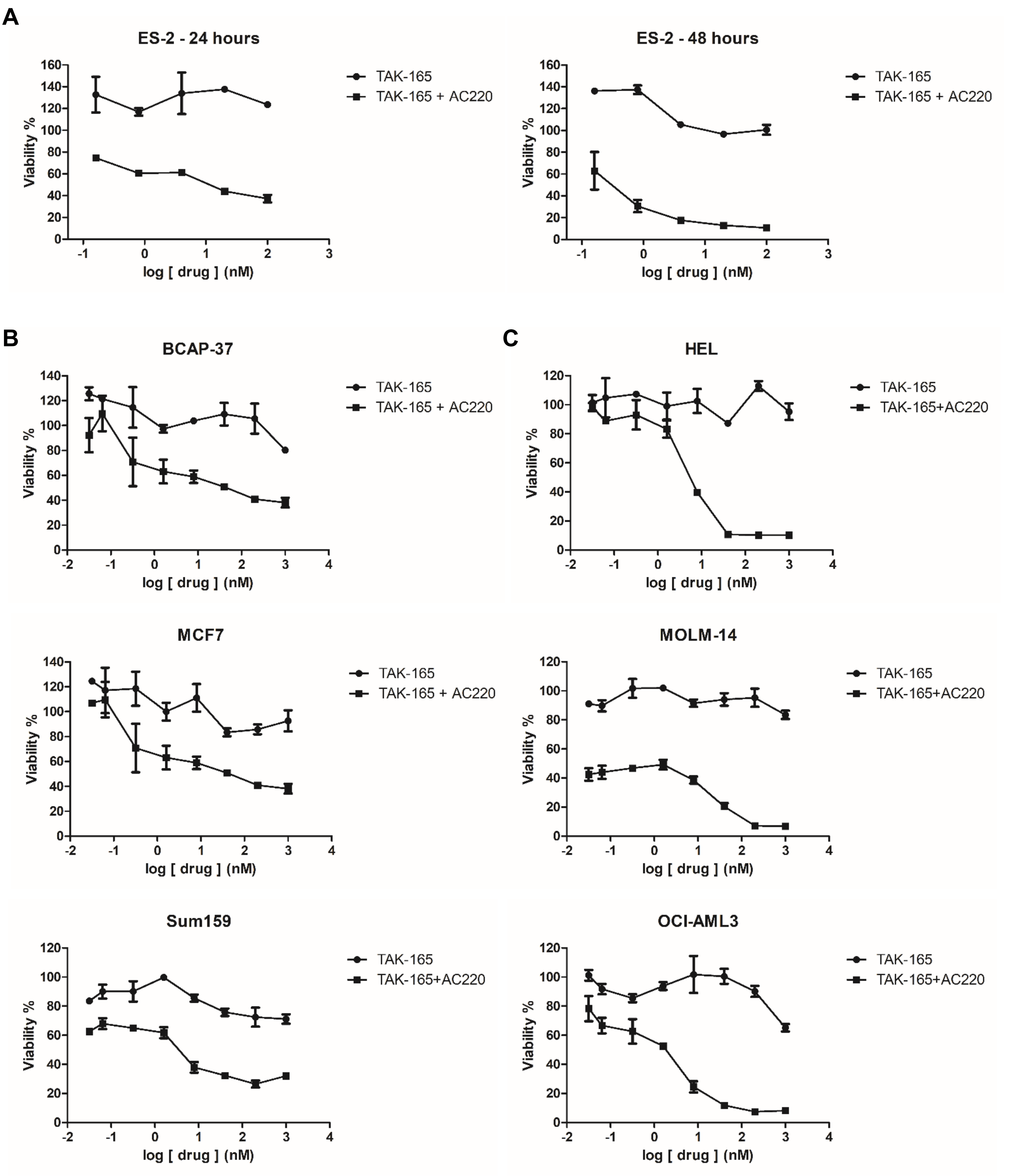

# Supplementary Figure 2

A

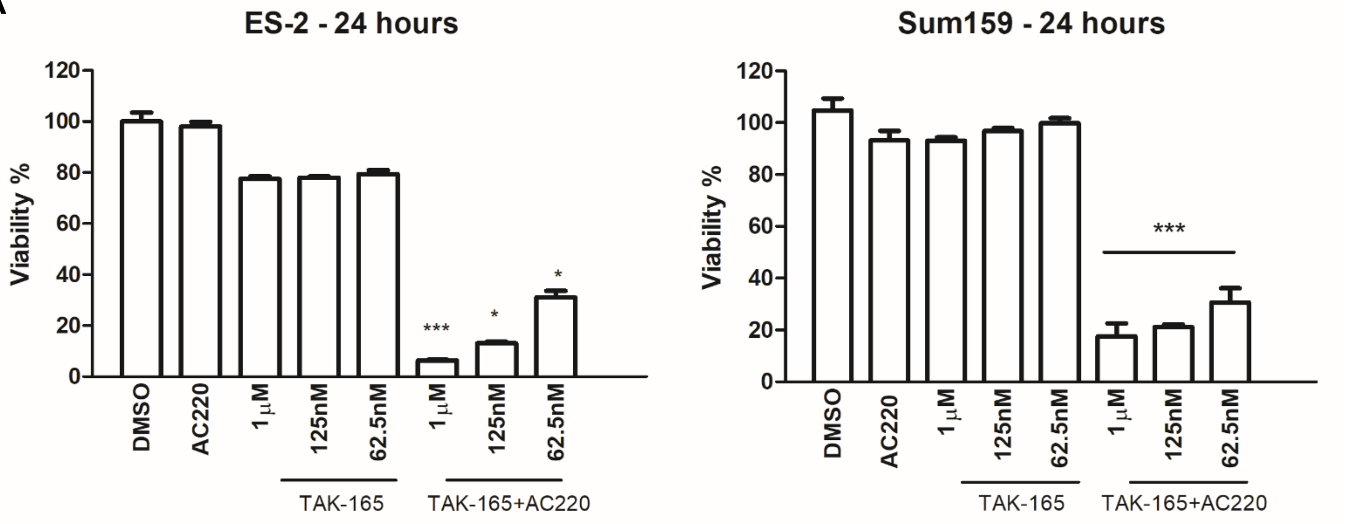

# Supplementary Figure 3

A

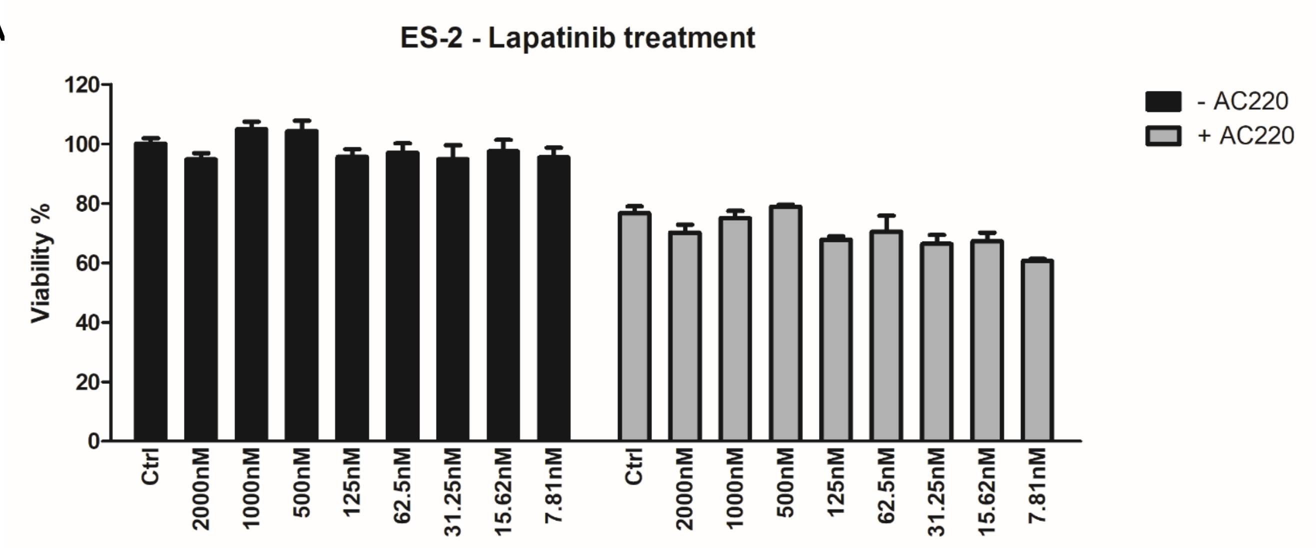

## Supplementary Figure 4

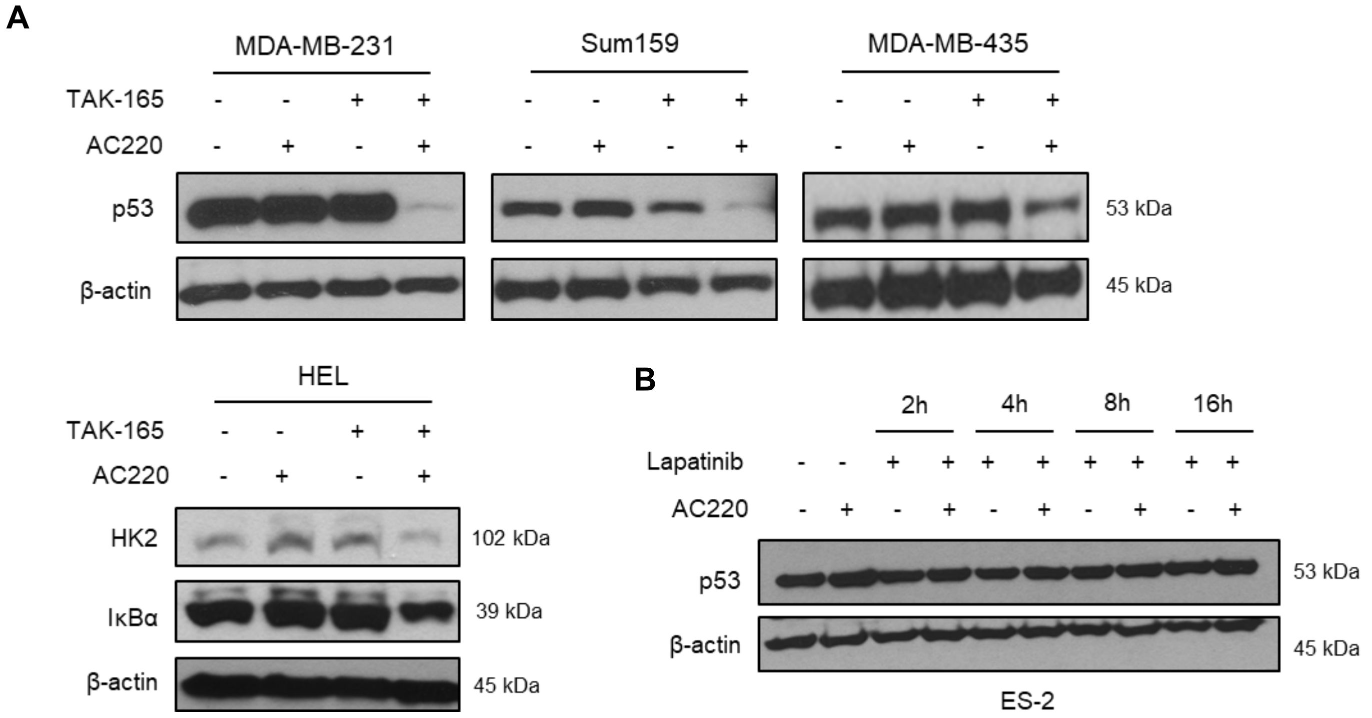

Supplement: Supplementary file 1 — Supplementary Figures 1, 2, 3 and 4 [file 41419_2017_170_MOESM1_ESM.pdf]
