# Supplementary material for: Synergistic effect of a novel autophagy inhibitor and Quizartinib enhances cancer cell death
Source: Cell Death Dis. 2018 Jan 26;9(2):138. doi: 10.1038/s41419-017-0170-9 (PMC5833862; doi:10.1038/s41419-017-0170-9)
Supplement: Supplementary file 2 — Supplementary Figures Legends [file 41419_2017_170_MOESM2_ESM.docx]

**Supplementary Figures Legends**

**Figure S1: The effect of TAK-165/AC220 combination on the viability of cancer cell lines are dose-dependent.** A) Cell viability (%) of ES-2 cells treated with TAK-165 at different concentrations and AC220 (2 μM) for 24 and 48 h. B) Cell viability (%) of breast cancer cells and AML cells (C) with TAK-165 at different concentrations and AC220 (2 μM) for 24 h. Viability was determined using Sulforhodamine B assay (Breast and ovarian cancer cells) and CellTiter-Glo^®^ Luminescent assay (AML cell lines). In the graphs, the first point of each curve belongs to TAK-165 (●) and TAK-165/AC220 (■) highest concentration treatments. Bars: Mean ± SD.

**Figure S2: TAK-165/AC220 combination induces cell death in cancer cells in a non-proliferative condition.** A) Cell viability (%) of confluent ES-2 and (B) Sum159 cells treated with TAK-165 at three different concentrations (1000, 125 and 62.5 nM) and AC220 (2 μM) for 24 h. Viability was determined using CellTiter-Glo^®^ Luminescent assay. In all the experiments, treatment groups were compared with control group, unless otherwise indicated. Bars: Mean ± SD. ***: *p* < 0.001.

**Figure S3: TAK-165 is a new autophagy inhibitor and its effect on cell death is HER2-independent.** A) Cell viability (%) of ES-2 cells treated with Lapatinib (HER2 inhibitor) at different concentrations and AC220 (2 μM) for 24 h. Viability was determined using CellTiter-Glo^®^ Luminescent assay. Anti-β-actin was used as a loading control. In all the experiments, treatment groups were compared with control group, unless otherwise indicated. Bars: Mean ± SD.

**Figure S4: TAK-165/AC220 combination activates chaperone-mediated autophagy in different cancer cell lines.** A) Immunoblotting of p53 levels in MDA-MB-231, Sum159 and MDA-MB-435 cells treated with TAK-165 (125 nM) and/or AC220 (2 μM) for 16 h. B) Immunoblotting of IκB-α and HK2 levels for HEL cells treated with TAK-165 (8 nM) and/or AC220 (1 μM) for 16 h. C) Lapatinib in combination with AC220 does not induce chaperone-mediated autophagy. Immunoblotting of p53 levels in ES-2 cells treated with Lapatinib (125 nM) and/or AC220 (2 μM) for 16 h. Anti-β-actin was used as a loading control.
